# Gut–Brain Axis and Neuroinflammation: The Role of Gut Permeability and the Kynurenine Pathway in Neurological Disorders

**DOI:** 10.1007/s10571-024-01496-z

**Published:** 2024-10-08

**Authors:** Rowan Kearns

**Affiliations:** Ulster University, Life and Health Sciences, Belfast, UK

**Keywords:** Gut permeability, Kynurenine pathway, Neurological disorders, Gut–brain axis, Neuroinflammation, Dysbiosis, Probiotic

## Abstract

The increasing prevalence of neurological disorders such as Alzheimer’s, Parkinson’s, and multiple sclerosis presents a significant global health challenge. Despite extensive research, the precise mechanisms underlying these conditions remain elusive, with current treatments primarily addressing symptoms rather than root causes. Emerging evidence suggests that gut permeability and the kynurenine pathway are involved in the pathogenesis of these neurological conditions, offering promising targets for novel therapeutic and preventive strategies. Gut permeability refers to the intestinal lining’s ability to selectively allow essential nutrients into the bloodstream while blocking harmful substances. Various factors, including poor diet, stress, infections, and genetic predispositions, can compromise gut integrity, leading to increased permeability. This condition facilitates the translocation of toxins and bacteria into systemic circulation, triggering widespread inflammation that impacts neurological health via the gut–brain axis. The gut–brain axis (GBA) is a complex communication network between the gut and the central nervous system. Dysbiosis, an imbalance in the gut microbiota, can increase gut permeability and systemic inflammation, exacerbating neuroinflammation—a key factor in neurological disorders. The kynurenine pathway, the primary route for tryptophan metabolism, is significantly implicated in this process. Dysregulation of the kynurenine pathway in the context of inflammation leads to the production of neurotoxic metabolites, such as quinolinic acid, which contribute to neuronal damage and the progression of neurological disorders. This narrative review highlights the potential and progress in understanding these mechanisms. Interventions targeting the kynurenine pathway and maintaining a balanced gut microbiota through diet, probiotics, and lifestyle modifications show promise in reducing neuroinflammation and supporting brain health. In addition, pharmacological approaches aimed at modulating the kynurenine pathway directly, such as inhibitors of indoleamine 2,3-dioxygenase, offer potential avenues for new treatments. Understanding and targeting these interconnected pathways are crucial for developing effective strategies to prevent and manage neurological disorders.

**Graphical Abstract:**

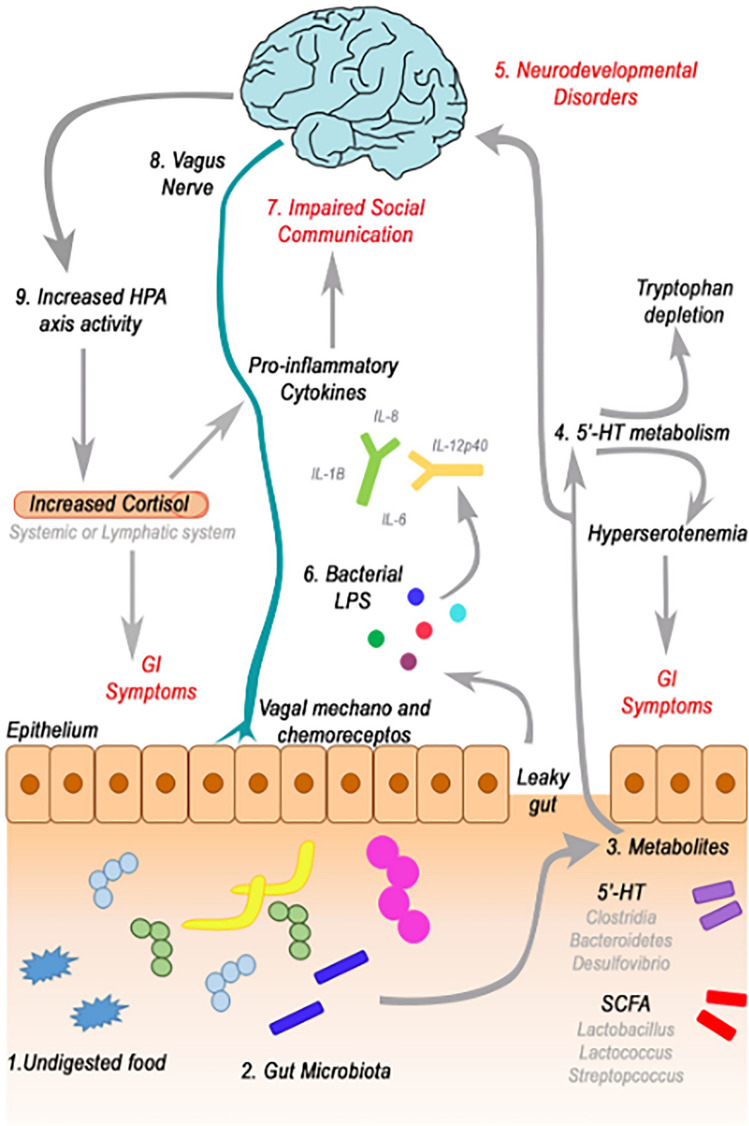

## Introduction

The rising prevalence of neurological disorders (NDs) such as Alzheimer’s disease (AD), Parkinson’s disease (PD), and multiple sclerosis (MS) poses a significant challenge to public health globally (Brown 2019). Despite extensive research, the exact mechanisms underlying these disorders remain elusive, with current treatments primarily focused on symptom management rather than addressing the root causes. Increasing evidence suggests that gut permeability, often referred to as “leaky gut”, and the kynurenine pathway (KP) plays crucial roles in the pathogenesis of these neurological conditions, presenting potential targets for novel preventive and therapeutic strategies (Erickson et al. 2012; Konsman 2022).

Gut permeability refers to the ability of the intestinal lining to prevent harmful substances from entering the bloodstream while allowing essential nutrients to pass through. A healthy gut barrier is critical for maintaining overall health, including neurological health (Allam-Ndoul et al. 2020). However, various factors such as poor diet, stress, infections, and genetic predispositions can compromise the integrity of the gut lining, leading to increased permeability. This condition allows toxins, bacteria, and other inflammatory substances to translocate from the gut into the systemic circulation, triggering widespread inflammation (Brown 2019).

The relationship between gut permeability and neurological disorders is mediated through the gut–brain axis (GBA), a complex communication network linking the gut and the central nervous system (CNS) (O’Mahony et al. 2015). This bidirectional pathway involves neural, hormonal, and immune signalling mechanisms, allowing the gut microbiota to influence brain function and vice versa. Dysbiosis, or an imbalance in the gut microbiota, can lead to increased gut permeability and systemic inflammation, which in turn can exacerbate neuroinflammation—a key factor in the development and progression of NDs (Dinan & Cryan 2017a, b).

One of the critical pathways implicated in this process is the KP, the primary route for tryptophan metabolism in the body (Mor et al. 2021). Under normal conditions, this pathway helps regulate immune responses and neurotransmitter production. However, in the context of increased gut permeability and systemic inflammation, the activation of the KP can become dysregulated. This dysregulation leads to the production of neurotoxic metabolites such as quinolinic acid, which can induce oxidative stress and excitotoxicity in the CNS, contributing to neuronal damage and the progression of neurological disorders (Hestad et al. 2022).

The KP’s involvement in neuroinflammation is particularly significant because it represents a potential point of intervention. By modulating this pathway, it may be possible to reduce the production of harmful metabolites and mitigate their effects on the brain. For instance, targeting the enzyme indoleamine 2,3-dioxygenase (IDO), which catalyses the initial step in the KP, could help reduce the levels of neurotoxic compounds and support neuronal health (Kennedy et al. 2017).

Moreover, the gut microbiota itself plays a crucial role in modulating the KP. Certain beneficial bacteria can influence the production of KP metabolites, thereby affecting neuroinflammatory processes. Probiotics and prebiotics, which help maintain a healthy gut microbiota, have shown promise in reducing gut permeability and systemic inflammation, potentially offering a protective effect against neurological disorders (Dinan et al. 2013; Sarkar et al. 2016; Xiang et al. 2022). These findings highlight the importance of maintaining a balanced gut microbiota through diet, lifestyle, and targeted interventions as a strategy for preventing and managing NDs (Miller et al., 2020).

In addition to dietary and microbial interventions, other preventive strategies may include pharmacological approaches aimed at modulating the KP directly. Research into specific inhibitors of enzymes involved in this pathway, such as IDO inhibitors, is ongoing and holds potential for developing new treatments for NDs. These inhibitors could help restore balance in the KP, reducing the burden of neurotoxic metabolites and supporting overall brain health (Platten et al. 2019).

### Microbiota and Gut Permeability

The concept of ‘microbiota’ originated in the early 1900s, recognising that a myriad of microorganisms, including bacteria, yeasts, and viruses, inhabit various human body sites such as the gut, skin, lungs, and oral cavity (Ursell et al. 2012). The precise timing of microbiota initiation, whether before or after birth, remains debated. Nevertheless, it is widely accepted that postnatal factors substantially influence the microbiome’s development (Matsuda et al. 2004). The GI tract, with its diverse microbial habitats, hosts the densest microbial population, comprising over 1500 bacterial species across more than 50 phyla (Dinan & Cryan 2017b). The predominant phyla include *Bacteroidetes* and *Firmicutes*, followed by *Proteobacteria*, *Fusobacteria*, *Tenericutes, Actinobacteria*, and *Verrucomicrobia*, accounting for approximately 90% of the microbial population (Louwies et al. 2020).

The intricate relationship between the immune system and the gut microbiota has evolved into a finely tuned mutualistic interaction essential for maintaining homeostasis. This balance ensures effective host immunity, preventing commensal microbes from exploiting resources excessively while maintaining tolerance to benign stimuli. However, various disruptions, such as antibiotic usage, dietary changes, and environmental pollutants, can destabilise this balance, leading to significant health implications. These disruptions can impair the interfaces between the host and microbiome, altering immune responses and potentially leading to systemic spread of commensal microbes, increased susceptibility to pathogenic invasion, and inappropriate immune reactions (Allam-Ndoul et al. 2020; Brown 2019; Morris et al. 2015).

**Fig. 1:**
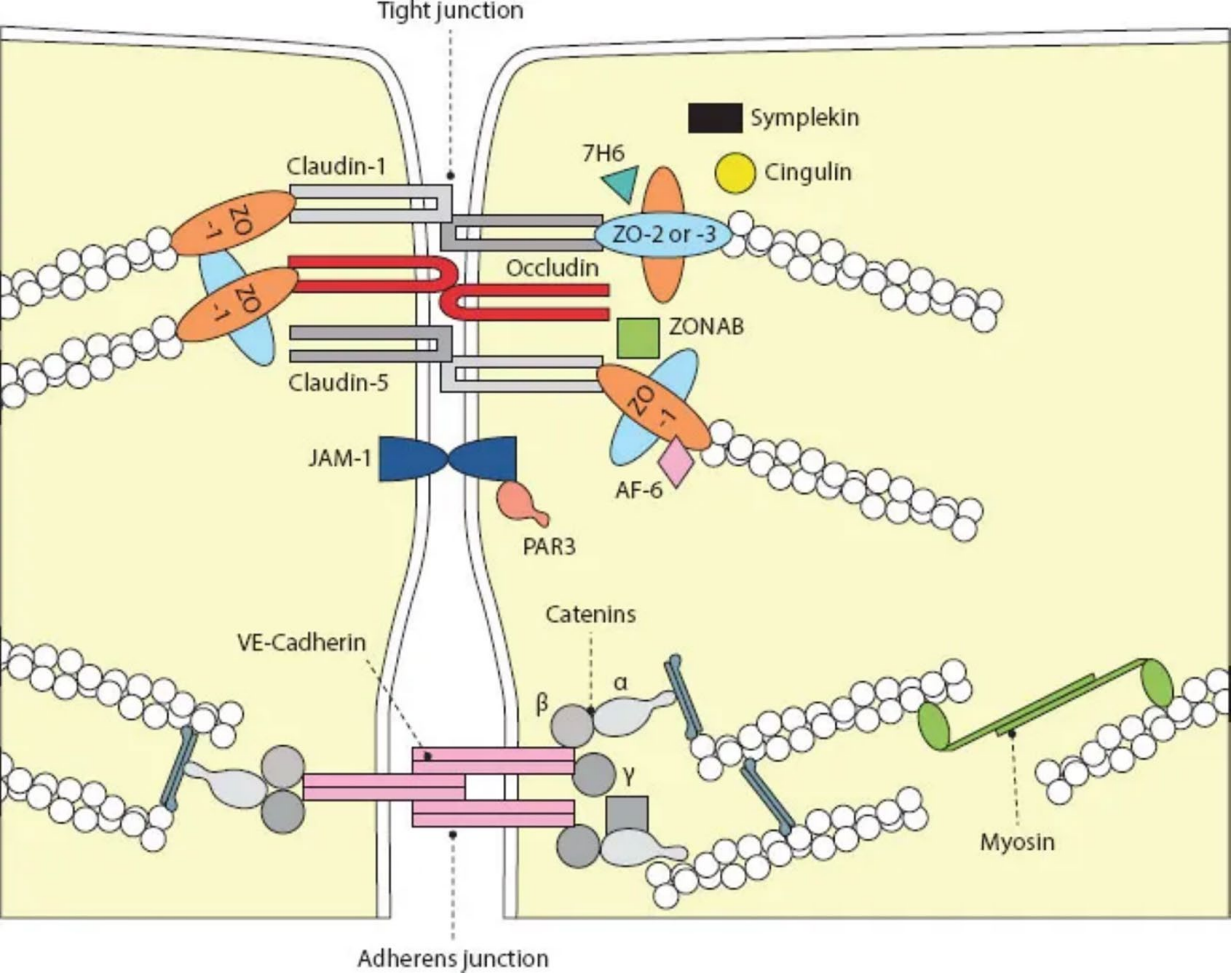
The molecular composition of the tight junction (TJ). TJs are constituted by the transmembrane proteins occludin, claudins, and junctional adhesion molecule 1 (JAM-1), which seal the paracellular space and connect TJ to the actin cytoskeleton via interaction with proteins from the zona occludens (ZO) family. (With kind permission from Springer Science + Business Media: Histochem. Cell Biol., Tight junctions and the modulation of barrier function in disease, 130(1), 2008, 55, Förster, C., Copyright 2008)

The gastrointestinal (GI) tract, a complex organ system responsible for nutrient digestion and absorption, also plays a critical role in maintaining a barrier that protects the body from harmful substances. The gut lining, or intestinal epithelium, is central to this function. It serves as a selectively permeable membrane, allowing essential nutrients to pass while preventing harmful pathogens and toxins from entering the body (Takiishi et al. 2017). The intestinal epithelial cells (IECs), primarily enterocytes, form the gut lining and regulate the trans-epithelial movement of substances. This lining is reinforced by a complex array of junction proteins, including tight junctions, adherens junctions, gap junctions, and desmosomes, which ensure the structural integrity and function of the gut barrier (Fig. 1) (Schoultz & Keita 2020; Wells et al. 2017). Tight junctions are key regulators of the paracellular pathway, controlling the flow of ions, water, and various macromolecules between epithelial cells (Suzuki 2020).

The gut microbiota employs various defence mechanisms to prevent pathogen overgrowth and resultant damage or infection. One such mechanism is colonisation resistance, where both commensal and pathogenic microorganisms compete for resources and functional space, often mediated by quorum sensing (Takiishi et al. 2017). IECs not only form a physical barrier but also engage in active immune responses. The mucus layer produced by goblet cells adds an additional protective layer, preventing direct contact between luminal bacteria and the epithelial surface (Tlaskalová-Hogenová et al. 2011).

The epithelial barrier is a multilayer system that provides both physical and functional protection. Key components include luminal intestinal alkaline phosphatase (IAP), which dephosphorylates bacterial endotoxin lipopolysaccharide (LPS) to detoxify it; the mucus layer, which acts as a physical barrier preventing interactions between gut bacteria and IECs; tight junctions, which limit the paracellular transport of bacteria and their products to systemic circulation; and antibacterial proteins and immunoglobulin A (IgA), secreted by Paneth cells and immune cells in the lamina propria, which contribute to mucosal defence(Iacob & Iacob 2019; Odenwald & Turner 2017).

The intestinal epithelium serves as an essential barrier, orchestrating selective permeability that balances nutrient absorption with the exclusion of harmful substances. This barrier function is meticulously regulated through transcellular and paracellular pathways (Fig. 1).

**Fig. 2:**
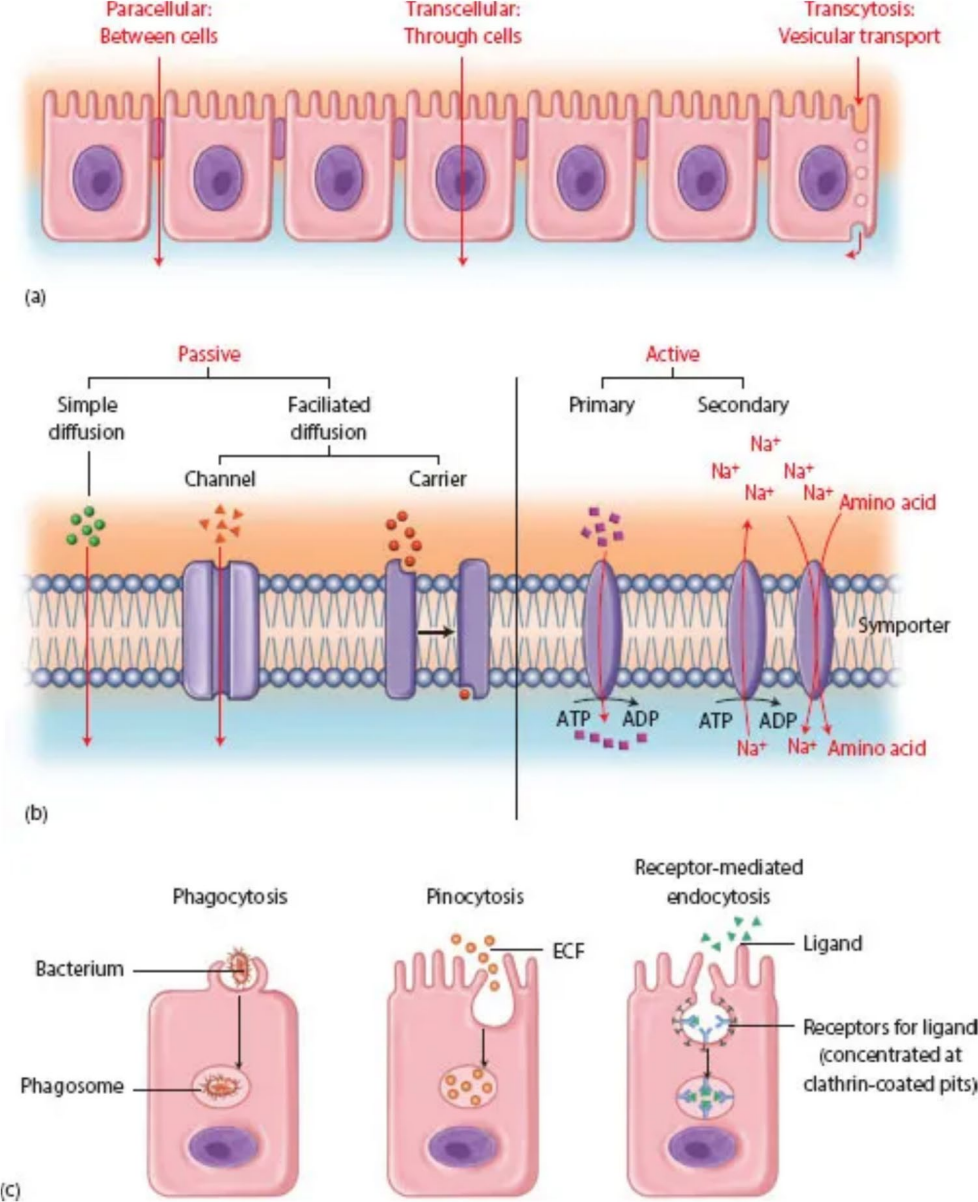
**a** General transport pathways: Paracellular, transcellular, and transcytosis. **b** Transcellular membrane transport: Transport across the apical cell membrane can be via (1) passive transport, which can be via (I) simple diffusion and (II) facilitated diffusion. Facilitated diffusion can, in turn, be either channel-mediated facilitated diffusion or carrier-mediated facilitated diffusion. (2) Active transport, which can be (I) primary active transport and (II) secondary active transport. **c** Endocytosis/transcytosis: Transport in vesicles that can be via (1) phagocytosis, via specialised cells of the reticuloendothelial system, e.g. neutrophils and macrophages, (2) pinocytosis: Nonspecific internalisation of extracellular fluid (ECF); any dissolved solutes that happen to be in the ECF also internalised and (3) receptor-mediated endocytosis/transcytosis, a highly selective type of endocytosis/transcytosis, by which cells take up specific ligands (Laurent et al. 2016)

The transcellular pathway involves the active transport of substances directly through epithelial cells, relying on various transporters and channels embedded in cell membranes. This route is highly specific and energy-dependent, facilitating the transport of nutrients, ions, and macromolecules (Madara 1998; Günzel & Yu 2013) (Fig. 2).

The paracellular pathway, on the other hand, allows passive movement of ions and small molecules between epithelial cells, regulated by tight junctions (Buckley & Turner 2018). This pathway is crucial for maintaining ionic balance and water flux in the intestinal lumen (Spadoni et al. 2015). Tight junctions are multi-protein complexes located at the apical region of epithelial cells, comprising transmembrane proteins such as claudins, occludins, and junctional adhesion molecules (JAMs), which interact with intracellular scaffold proteins like zonula occludens (ZO-1, ZO-2, ZO-3), linking tight junctions to the actin cytoskeleton (Odenwald & Turner 2017; Okumura & Takeda 2017).

The pore pathway within tight junctions allows the passage of small ions and uncharged molecules, regulated primarily by claudins forming charge-selective pores (Sturgeon & Fasano 2016). The leak pathway, which is less selective, accommodates the passage of larger molecules, including proteins and larger ions, regulated by occludin and ZO proteins (Odenwald & Turner 2017). Transepithelial resistance measures the epithelium’s ability to resist ion flow, serving as an indicator of paracellular permeability, with low TER values indicating high permeability (Schoultz & Keita 2020).

Intestinal permeability is dynamically regulated by various physiological and pathological factors. Inflammatory mediators such as pro-inflammatory cytokines (e.g. Tumour Necrosis Factor-alpha and Interferon-gamma) can disrupt tight junctions, increasing paracellular permeability. These cytokines activate signalling pathways leading to the reorganisation of tight-junction proteins and the actin cytoskeleton, resulting in increased permeability, particularly relevant in inflammatory conditions such as inflammatory bowel disease (IBD) (Nishida et al. 2018; Sturgeon & Fasano 2016).

Myosin light chain kinase (MLCK) also plays a crucial role in regulating tight-junction permeability. Activation of MLCK leads to the phosphorylation of myosin light chains, causing contraction of the actomyosin ring and increasing tight-junction permeability, a mechanism involved in conditions associated with increased intestinal permeability, such as chronic stress and infection (Buckley & Turner 2018). Dietary components and the gut microbiota significantly influence intestinal permeability. Short-chain fatty acids (SCFAs), produced by the fermentation of dietary fibres by gut bacteria, enhance barrier function by modulating the expression of tight-junction proteins, whereas high-fat diets and dysbiosis can disrupt tight-junction integrity, leading to increased permeability (Lee et al. 2018).

Oxidative stress, induced by reactive oxygen species (ROS), can damage tight-junction proteins, leading to increased permeability (Mor et al. 2021). Antioxidant mechanisms are essential for maintaining tight-junction integrity and protecting against oxidative stress-induced barrier dysfunction, particularly relevant in conditions such as IBD and metabolic disorders (Bailey et al. 2011). Hormones and neuropeptides, such as glucocorticoids and corticotropin-releasing hormone, also modulate intestinal permeability, with stress-induced release of these hormones leading to increased permeability, highlighting the intricate connection between the gut and the CNS (Zuhl et al. 2015).

### The Microbiota–Gut–Brain Axis

The GBA is a bidirectional communication network orchestrated through a complex network of neurons, neurotransmitters, hormones, and immune mediators. While its significance in mental and cognitive health is widely acknowledged, the precise mechanisms through which the gut microbiota exerts influence on brain development and function remain an active area of investigation (Berding et al. 2021; Breit et al. 2018; Dinan & Cryan 2017b; Gomez-Eguilaz et al. 2019; Louwies et al. 2020). The GBA encompasses a multifaceted communication network primarily between the central and enteric nervous systems, linking the brain’s emotional and cognitive centres with intestinal function (O’Mahony et al. 2015). Both clinical and experimental research underscore that dysfunctions along the GBA could be implicated in brain and cognitive diseases (Dinan & Cryan 2017b). Alterations in the gut microbiota can modulate the peripheral and CNS, influencing brain stimulation and cognitive functioning via various signalling pathways (Dinan & Cryan 2017a).

Neuronal pathways form a critical component of the GBA. The vagus nerve, extending from the brainstem to the gut, is pivotal in these pathways (Dinan & Cryan 2017b). It detects sensory signals from the gut and conveys them to the CNS, involving the activation of mechanoreceptors and chemoreceptors responsive to chemical stimuli, including hormones, neurotransmitters, and metabolites produced by EECs (O’Mahony et al. 2015). The ENS, often referred to as the “second brain”, contains an extensive neuronal network regulating gut functions and is influenced by the gut microbiota, impacting gut motility and intestinal barrier function (Zhu et al. 2024).

Both the CNS and the gut microbiota directly affect, and are affected by, the immune system. The gut microbiota is crucial in modulating the development and function of the peripheral immune system (C. Wang et al. 2019). It is also integral to the healthy development, maturation, and activation of microglia, which are the innate immune cells in the brain (Erny et al. 2015). The activation of microglia is believed to depend on signals from microbial metabolism, as evidenced by the restoration of microglial morphology and function in germ-free mice treated with bacterial derived (Erny et al. 2015).

The gut microbiota’s interaction with the brain is also mediated through the systemic immune system via circulating cytokines (Campbell et al. 2014). Changes in systemic immunity can lead to altered immune signalling within the brain, resulting in symptoms such as loss of appetite, irritability, low mood, loss of motivation, social withdrawal, fatigue, and impaired attention (Anderson et al. 2021; Chen et al. 2021; Connell et al. 2017; Huang & Wu 2021). Cytokines and chemokines produced by brain-resident immune cells are transported directly across the blood–brain barrier (BBB) and play a significant role in this interaction (M.M.A. et al. 2016). There is evidence suggesting that the gut microbiota influences BBB permeability, as studies have shown that germ-free mice exhibit increased BBB permeability, partly due to reduced expression of tight-junction proteins such as occludin and claudin 5 (Braniste et al. 2014). The microbiota is vital in the initial maturation of the immune system, influencing the expression of toll-like receptors (TLRs) on immune cells and guiding the development of antigen-specific acquired immunity (O’Hara & Shanahan 2006). The hypothesis that alterations in gastrointestinal permeability may trigger systemic inflammatory responses is supported by findings of increased levels of lipopolysaccharides (LPS) and corresponding immunoglobulins in conditions such as depression, autism, and Alzheimer’s disease (Asanka Sanjeewa et al. 2020; Ghosh et al. 2020; Millischer et al. 2021).

### Dysbiosis

Dysbiosis refers to the disruption of the delicate balance between the host and its gut microbiota, which can lead to adverse health effects (Levy et al. 2017). Dysbiosis can be triggered by various factors, including antibiotic use, dietary changes, environmental pollutants, and infections, compromising the integrity of the gut barrier, altering immune responses, and resulting in systemic inflammation (Iacob & Iacob 2019; Martinez et al. 2021).

Antibiotic use is a primary driver of dysbiosis, as antibiotics eliminate beneficial microbes, leading to a significant reduction in microbial diversity and the overgrowth of resistant strains (Fröhlich et al. 2016). Dietary changes, particularly those involving high fat and sugar intake, promote the growth of harmful bacteria while reducing beneficial populations, disturbing the microbial balance in the gut (Sonnenburg & Sonnenburg 2019). Environmental pollutants, such as heavy metals and pesticides, can directly affect microbial populations or alter the host’s physiology, further destabilising the gut microbiome (Rinninella et al. 2019). Pathogenic infections outcompete commensal bacteria for resources and niches, triggering inflammatory responses that disrupt the microbial community (C. Wang et al. 2019).

Dysbiosis impairs the interfaces between the host and the microbiome, leading to increased intestinal permeability, immune dysregulation, and systemic inflammation. The disruption of gut barrier integrity often allows harmful substances, including LPS from Gram-negative bacteria, to translocate from the gut lumen into the systemic circulation (Ghosh et al. 2020). LPS, a major component of the outer membrane of Gram-negative bacteria, is a potent activator of the inflammatory response and is recognised by TLRs on immune cells, leading to the production of pro-inflammatory cytokines (Brown 2019).

Dysbiosis can lead to inappropriate immune responses, including increased susceptibility to infections, autoimmune diseases, and chronic inflammatory conditions (Levy et al. 2017). The loss of beneficial microbial populations that produce anti-inflammatory compounds, such as SCFAs, exacerbates immune dysregulation (Sonnenburg & Sonnenburg 2019). The translocation of endotoxins like LPS into the systemic circulation triggers an inflammatory response (explained below). Chronic low-grade inflammation resulting from endotoxemia is associated with various diseases, including metabolic disorders, cardiovascular diseases, and neuroinflammatory conditions (Park et al. 2023).

The inflammatory response to endotoxins significantly impacts multiple pathways. Toll-Like Receptor 4 (TLR4) signalling, for example, recognises LPS and initiates a cascade of immune responses. Upon LPS binding, TLR4 dimerises and recruits adaptor proteins such as Myeloid differentiation primary response 88 (MyD88) and TIR-domain-containing adapter-inducing interferon-β (TRIF), leading to the activation of nuclear factor kappa-light-chain-enhancer of activated B cells (NF-κB) and the production of pro-inflammatory cytokines, including interleukin-6 (IL-6), tumour necrosis factor-alpha (TNF-α), and interleukin-1 beta (IL-1β) (Asanka Sanjeewa et al. 2020; Hunter & Jones 2015). This pathway is crucial for mounting an effective immune response but can lead to chronic inflammation if dysregulated (Campbell et al. 2014).

The Nucleotide-binding oligomerisation domain, Leucine Rich Repeat and Pyrin domain-containing 3 (NLRP3) inflammasome is a key component in the body’s inflammatory response (Plaza-Díaz et al. 2017). Dysbiosis can activate the NLRP3 inflammasome through microbial products like LPS, leading to the activation of caspase-1 and the maturation of interleukin-1 beta (IL-1β) and interleukin-18 (IL-18). This pathway, while essential for responding to infections, can contribute to inflammatory diseases if overactivated (Guo et al. 2023).

The Janus Kinase-Signal Transducer and Activator of Transcription (JAK-STAT) pathway, activated by pro-inflammatory cytokines like IL-6, also contributes to inflammatory responses. Binding of these cytokines to their receptors leads to the phosphorylation of JAKs, which then phosphorylate STATs. Phosphorylated STATs dimerise and translocate to the nucleus to induce the expression of inflammatory genes, playing a significant role in inflammatory and autoimmune diseases (Asanka Sanjeewa et al. 2020).

The Mitogen-Activated Protein Kinase (MAPK) pathway, another critical signalling mechanism activated by TLRs and cytokine receptors, involves a cascade of protein kinases that lead to the transcription of inflammatory mediators. Dysbiosis-induced activation of the MAPK pathway contributes to the production of cytokines and other inflammatory responses, highlighting its role in chronic inflammation and disease (Sturgeon & Fasano 2016).

Among the pathways activated by dysbiosis-induced inflammation, the KP holds particular significance due to its implications in neurological disorders. The KP is a major route for tryptophan metabolism, producing neuroactive metabolites that significantly impact brain function and fatigue perception. In conditions like Alzheimer’s disease and major depressive disorder, dysregulation of this pathway leads to increased production of neurotoxic metabolites such as quinolinic acid, resulting in neuroinflammation and neurodegeneration (Chatterjee et al. 2018; Marx et al. 2021). This link between the KP and neurological disorders suggests that targeting this pathway may offer therapeutic potential.

### Kynurenine Pathway

**Fig. 3:**
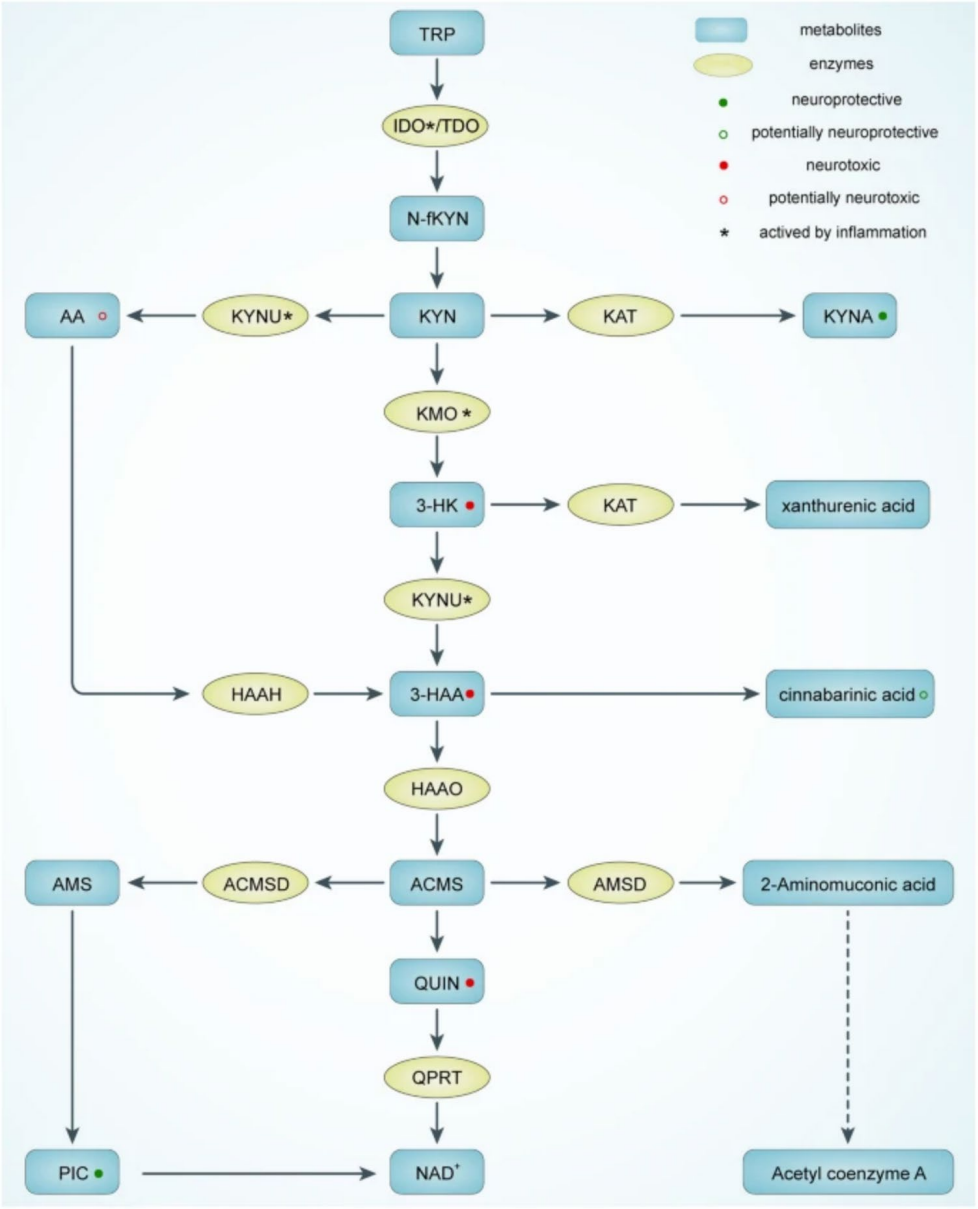
The tryptophan-kynurenine metabolic pathway. *TRP* tryptophan; *IDO* indoleamine 2,3-dioxygenase; *TDO* tryptophan 2,3-dioxygenase; *N-fKYN* N-formyl-kynurenine; *AA* anthranilate acid; *KYNU* kynureninase; *KYN* kynurenine; *KAT* kynurenine aminotransferase; *KYNA* kynerunic acid; *KMO* kynurenine 3-monooxygenase; *3-HK* 3-hydroxykynurenine; *HAAH* 3-hydroxyanthranilic acid 3,4-hydroxylase; *3-HAA* 3-hydroxyanthranilic acid; *HAAO* 3-hydroxyanthranilicacid 3,4-dioxygenase; *AMS* 2-aminomuconic-6-semialdehyde; *ACMSD* 2-amino-3-carboxymuconate-6-semialdehydedecarboxylase; *ACMS* 2-amino-3-carboxymuconate-6-semialdehyde; *AMSD* 2-aminomuconic-6-semialdehyde dehydrogenase; *QUIN* quinolinic acid; *QPRT* quinolinate phosphoribosyltransferase; *PIC* picolinic acid; *NAD + * nicotinamide adenine dinucleotide

The KP begins with the conversion of tryptophan (TRP) to N-formylkynurenine (N-fKYN), a reaction catalysed by the rate-limiting enzymes indoleamine 2,3-dioxygenase (IDO) and tryptophan 2,3-dioxygenase (TDO). N-fKYN is then hydrolysed to kynurenine (KYN) by kynurenine formamidase. TDO primarily facilitates basal TRP metabolism in the liver, whereas IDO is predominantly active in immune cells and can be induced by pro-inflammatory cytokines such as interferon-gamma (IFN-γ), interleukin-1 (IL-1), IL-6, and tumour necrosis factor-alpha (TNF-α) (Fig. 3) (Chen et al. 2021; Hestad et al. 2022).

KYN is a crucial metabolite within the pathway, branching into several significant metabolites. One branch converts KYN into kynurenic acid (KYNA) via the enzyme kynurenine aminotransferase (KAT). KYNA is recognised for its neuroprotective properties, acting as an antagonist to the N-methyl-D-aspartate (NMDA) receptor. Another branch converts KYN into anthranilic acid (AA) through the action of kynureninase (KYNU). A third branch transforms KYN into 3-hydroxykynurenine (3-HK) via kynurenine 3-monooxygenase (KMO) (Fig. 3) (Schwartz 2014).

3-HK can be further metabolised into 3-hydroxyanthranilic acid (3-HAA) by KYNU or into xanthurenic acid (XA) by KAT. In the brain, AA is efficiently converted into 3-HAA, which can subsequently form cinnabarinic acid (CA) or 2-amino-3-carboxymuconate-6-semialdehyde (ACMS). ACMS has several metabolic fates: It can be converted to quinolinic acid (QUIN), an NMDA receptor agonist and neurotoxin, or to picolinic acid (PIC) via 2-amino-3-carboxymuconate-6-semialdehyde decarboxylase (ACMSD). QUIN is particularly noteworthy as it can further metabolise into nicotinamide adenine dinucleotide (NAD +) via quinolinate phosphoribosyltransferase (QPRT), highlighting the pathway’s role in cellular energy metabolism (Anderson et al. 2021; Kennedy et al. 2017).

### Gut-Derived Inflammation and Its Role in Neuroinflammation

The activation of the KP by pro-inflammatory cytokines links peripheral inflammation to CNS effects. Dysbiosis-induced endotoxemia, through the activation of TLR4 and subsequent cytokine release, can influence the KP. Elevated levels of pro-inflammatory cytokines such as TNF-α, IL-6, and IL-1β in response to LPS translocation can enhance IDO activity, skewing tryptophan metabolism towards neurotoxic pathways. Furthermore, the metabolites of the KP, particularly QUIN, contribute to the neuroinflammatory milieu. QUIN activates microglia, the resident immune cells of the CNS, further propagating inflammatory responses within the brain. This creates a feedback loop where peripheral inflammation exacerbates central inflammation, potentially leading to or worsening neurological disorders (Calcia et al. 2016; Erickson et al. 2012).

### Pathways Leading to Kynurenine Pathway Activation

The inflammatory response induced by endotoxin involves the activation of TLR4 with its co-receptor Myeloid Differentiation factor 2 (MD2) on immune cells. This interaction initiates intracellular signalling cascades involving MyD88, TRAF6, and the IκB kinase (IKK) complex, leading to the activation of NF-κB. NF-κB translocates into the nucleus, upregulating genes encoding pro-inflammatory cytokines such as IL-6 and TNF-α (Lawrence 2009; Nishida et al. 2018; Wu et al. 2018). IL-6 and TNF-α exert their effects by binding to their respective receptors, IL-6R and TNFR1/2, on target cells. IL-6 primarily activates the JAK-STAT pathway, leading to the phosphorylation and activation of STAT3, which regulates inflammatory gene transcription. TNF-α activates various signalling pathways, including NF-κB, Jun N-terminal kinase (JNK), and Mitogen-activated protein kinases (MAPK) pathways. In addition, IFN-γ upregulates the enzyme IDO, catalysing the conversion of tryptophan into N-formylkynurenine, initiating the KP. KMO further converts kynurenine into 3-hydroxykynurenine, an intermediate in the QUIN pathway (Kennedy et al. 2017; Köhler et al. 2021). Pro-inflammatory cytokines like IFN-γ can stimulate KMO, potentially promoting neurotoxicity by favouring the QUIN pathway (Hestad et al. 2022). These inflammatory signals can affect neural drive, contributing to feelings of sadness and increased perceptions of fatigue, potentially leading to neuroinflammatory and neuropsychiatric conditions (Felger 2017; Yamashita 2020). Neuroinflammation mediated by the KP is implicated in the pathogenesis of various neurological disorders, including depression, Alzheimer’s disease, and Parkinson’s disease (Bay-Richter & Wegener 2022; Megur et al. 2021; Venkatesan et al. 2020).

### Cytokine Transmission to the Brain

Increased levels of cytokines in the periphery can reach and affect the brain through several mechanisms. These include passage through leaky regions in the BBB such as circumventricular organs, active transport through transport molecules, activation of cells lining the cerebral vasculature (endothelial cells and perivascular macrophages), binding to cytokine receptors associated with the vagus nerve, stimulating the hypothalamic–pituitary–adrenal (HPA) axis at the anterior pituitary or hypothalamus, and recruitment of activated cells such as monocytes/macrophages from the periphery to the brain (Bhatt et al. 2023; Konsman 2022; Kvichansky et al. 2019). Through activation of the intracellular signalling MAPK pathway, cytokines can increase the number and function of the reuptake pumps for serotonin, noradrenaline, and dopamine, which in turn can reduce the availability of these neurotransmitters within the synaptic cleft. Preclinical studies have demonstrated that increased inflammatory cytokines reduce central levels of brain-derived neurotrophic factor (BDNF) and neurogenesis, leading to depressive-like behaviour (Dantzer 2009). However, the relationship between peripheral and central inflammatory markers and antidepressants is complex, and it remains unclear which pathways are most relevant for cytokine signal transmission in stress-related disorders such as depression (Bhatt et al. 2023; Felger 2017; Huang & Wu 2021).

### Anti-inflammatory Agents and Depression

There is some evidence, albeit from small studies of short duration, suggesting that anti-inflammatory agents such as non-steroidal anti-inflammatory drugs (NSAIDs) and cytokine inhibitors reduce depressive symptoms (Bhatt et al. 2023). For depressed patients with raised inflammatory markers, this raises the prospect of whether reducing low-grade inflammation could alleviate depressive symptoms. Although a randomised controlled trial of the monoclonal antibody infliximab, a TNF-α antagonist, was not superior to placebo in reducing depressive symptoms overall, in patients with high baseline CRP levels there were greater reductions in depressive symptoms than in those with low CRP levels(Liu et al. 2022). Another study showed that CRP level at baseline differentially predicted treatment outcome with escitalopram or nortriptyline (Fourrier et al. 2018). These studies provide the impetus for stratification of depressed patients based on inflammatory profiles to advance personalised medicine. Developing more nuanced profiles of inflammatory proteins and gene expression, as well as cellular immune parameters, likely represents the future for predictors and targets of response to anti-inflammatory therapies.

Reductions in basal ganglia activity have been noted in more posterior regions, where they are associated with fatigue, and in more ventral regions (such as the nucleus accumbens), where they have been linked to the development of anhedonia(Capuron & Miller 2011; Felger 2017). These areas are crucial for motivation and reward processing, and their impairment can lead to the core symptoms of depression, such as lack of pleasure in activities (anhedonia) and reduced motivation.

### The Role of Microglia in Neuroinflammation

Microglia, the resident immune cells of the CNS, are central to the inflammatory process and a source of cytokines. These phagocytic cells account for approximately 10% of cells in the brain and contribute to the plasticity of neural circuits by modulating synaptic architecture and function (Calcia et al. 2016; Erny et al. 2015). Microglial process motility can be modulated by glutamatergic and GABAergic neurotransmission. Acute stress results in microglia activation and increased levels of pro-inflammatory cytokines in areas such as the hippocampus and hypothalamus (Coxon et al. 2018). Most studies show increases in activated microglia in response to chronic stress. Preliminary changes in the microenvironment of the microglia may result in susceptibility to secondary inflammatory stimuli. This concept of microglia priming may be relevant to depression, which often requires multiple environmental “hits” (Setiawan et al. 2015).

In an environmental two-hit rodent model, the first experimental manipulation targeted pregnant dams, and the second manipulation was given to the resulting offspring. Exposure to prenatal immune challenge and peripubertal stress synergistically induced pathological effects on adult behavioural functions and neurochemistry (Giovanoli et al. 2013). Early-life stress primes microglia, leading to a potentiated response to subsequent stress. The microbiota regulates microglia maturation and function, further underscoring the gut–brain axis’s role in neuroinflammation.

Clinically, microglial activation in the prefrontal cortex (PFC), anterior cingulate cortex (ACC), and insula in medication-free depressed patients has been demonstrated using translocator protein density measured by distribution volume in a positron emission tomography study (Deng et al. 2023; Setiawan et al. 2015). This evidence suggests that microglial activation is a significant contributor to neuroinflammation in depression and other neuropsychiatric conditions.

### Implications for Neurological Disorders

The chronic activation of the KP can lead to sustained production of neurotoxic metabolites like QUIN, contributing to neurodegeneration and cognitive decline (Hestad et al. 2022). In Alzheimer’s disease, for example, the accumulation of neurotoxic KP metabolites can exacerbate amyloid-beta and tau pathologies, driving disease progression (Guillemin & Brew 2002; Onyango et al. 2021).

In Parkinson’s disease, the dopaminergic neurons in the substantia nigra are particularly vulnerable to KP metabolites, leading to motor symptoms and further neuronal loss (Venkatesan et al. 2020). The role of the KP in these diseases highlights the need for therapeutic strategies targeting this pathway to mitigate neuroinflammation and neurodegeneration.

### Therapeutic Potential of Modulating the Kynurenine Pathway

Targeting the KP offers a promising therapeutic strategy for managing neuroinflammatory and neurodegenerative disorders. Inhibitors of IDO and KMO, for instance, can reduce the production of neurotoxic metabolites and shift the balance towards neuroprotective metabolites like kynurenic acid. Preclinical studies have shown that such inhibitors can attenuate neuroinflammation and protect against neuronal damage in models of Alzheimer’s and Parkinson’s diseases (Atlam et al. 2018; Campbell et al. 2014).

### Neurological Diseases

#### Kynurenine Association with Neurological Conditions

Alzheimer’s disease (AD) is characterised by cognitive and memory deficits, amyloid plaques, tau tangles, neuroinflammation, synapse loss, and neuronal loss. Research indicates a significant association between AD and endotoxin levels, with mean blood endotoxin levels in AD patients increased threefold, and brain endotoxin levels elevated two- to threefold. Endotoxin, found within amyloid plaques, can promote amyloid-beta production and aggregation as well as tau hyperphosphorylation. Studies have shown that eliminating gut bacteria reduces plaque load and microglial activation in amyloid model mice (Bello-Medina et al. 2022; K. et al. 2017).

The KP, a major route of tryptophan metabolism, plays a crucial role in AD. Dysregulation of the KP has been observed in AD, leading to increased levels of neurotoxic metabolites and contributing to neuroinflammation and neurodegeneration (Fathi et al. 2022; Guillemin & Brew 2002). Studies have shown altered levels of KP metabolites in the cerebrospinal fluid (CSF) and blood of AD patients, indicating the pathway’s involvement in the disease’s pathogenesis (Si et al. 2023).

Genetic risk for AD is closely linked to APOE isoforms, with APOE2 being protective, APOE3 neutral, and APOE4 detrimental. LPS injection strongly induces serum ApoE in rodents, and ApoE binds LPS, facilitating its uptake and degradation by the liver. APOE4 variant carriers are more sensitive to LPS toxicity than those with APOE3. Variants in the LPS-receptor TLR4 and the LPS-binding receptor TREM2 are also associated with increased AD risk, further establishing the genetic connection between AD and endotoxin (Ghosh et al. 2020; Millischer et al. 2021).

Parkinson’s Disease (PD) is marked by motor dysfunctions, α-synuclein aggregates (Lewy bodies), and dopaminergic neuron loss in the substantia nigra (Kalia & Lang 2015). PD patients often exhibit gut dysfunction and increased gastrointestinal permeability. A significant number of PD patients have elevated blood endotoxin levels, and their gut microbiome differs from that of controls. The gut microbiome’s influence on motor symptoms in PD is evidenced by studies showing that eliminating gut bacteria prevents motor deficits, while introducing the microbiome from PD patients worsens pathology. Colonisation with endotoxin-producing Helicobacter pylori is associated with PD, and its eradication improves symptoms (Lin et al. 2019; Park et al. 2023).

In mice, a single dose of peripheral endotoxin increases α-synuclein expression in intestinal neurons and permeability, mirroring PD patients. Endotoxin also promotes α-synuclein production and fibrillisation. Peripheral endotoxin injection in wild-type and transgenic mice (expressing human A53T mutant α-synuclein) causes acute neuroinflammation, but only transgenic mice develop persistent neuroinflammation, aggregated α-synuclein, and progressive dopaminergic neuron degeneration. This supports the dual-hit hypothesis for PD: endotoxin exposure combined with aggregable α-synuclein leads to neurodegeneration. Nonetheless, endotoxin alone can induce dopaminergic neuron loss in the substantia nigra in mice after several months (Brown 2019; Earls et al. 2019).

The KP is also implicated in PD. Changes in gut microbiota composition have been linked with altered neurotransmitter levels and increased inflammation, potentially contributing to PD progression. The KP of tryptophan catabolism is a key regulator of the immune response and is involved in inflammatory and neurotoxic processes in Parkinson’s disease. Elevated levels of neurotoxic KP metabolites such as QUIN are observed in PD, contributing to neuronal damage and inflammation (Lin et al. 2019; Xiang et al. 2022).

Motor neuron disease, including amyotrophic lateral sclerosis (ALS), involves neurodegeneration of motor neurons and shares genetic and pathological mechanisms with frontotemporal dementia (FTD). Elevated blood endotoxin levels in ALS patients may result from gut inflammation and microbiome alterations. In vitro studies show that LPS addition to microglia or astrocytes causes TDP-43 mis-localisation and aggregation (Quek et al. 2022). Similarly, peripheral LPS administration to TDP-43(A315T) transgenic mice results in TDP-43 aggregation in vivo, suggesting a dual-hit hypothesis for ALS and FTD, where increased endotoxin levels combined with aggregable TDP-43 lead to neurodegeneration(Bright et al. 2021; Correia et al. 2015).

### Preventative Measures

The integrity of the intestinal barrier is crucial in preventing the onset and progression of inflammatory diseases. Various strategies have been developed to enhance gut barrier function, addressing both genetic and environmental factors that can compromise intestinal integrity.

Larazotide Acetate is one promising therapeutic agent that inhibits the protein modulator zonulin, which regulates intestinal permeability by modulating TJ disassembly. In coeliac patients, Larazotide Acetate has demonstrated efficacy in reducing disease symptoms by preventing the breakdown of these junctions, thus improving gut barrier function (Leffler et al. 2015). By maintaining the integrity of the intestinal barrier, this drug helps to prevent the translocation of harmful substances, which can trigger systemic inflammation and contribute to various diseases.

Tofacitinib, an inhibitor of inflammatory enzymes associated with rheumatoid arthritis, has shown potential in preventing cytokine-induced gut barrier dysfunction. It works by limiting the delocalisation of TJ proteins such as ZO-1 in intestinal epithelial cells (IECs) exposed to IFN-γ. This action makes it a promising therapeutic agent for inflammatory bowel disease (IBD), where elevated IFN-γ levels in the inflamed intestinal tissues contribute to barrier dysfunction (Spalinger et al. 2021). This drug’s ability to preserve the integrity of the gut barrier highlights its potential in mitigating chronic inflammation associated with IBD (Cleynen et al. 2016).

Another therapeutic approach involves potassium-competitive acid blockers like Tegoprazan, which offer a promising alternative for treating gastric acid-related diseases and protecting against IBD development. Tegoprazan enhances gut barrier health by increasing the abundance of anti-inflammatory bacteria such as *Bacteroides vulgatus*. These beneficial bacteria help to limit the adhesion of pathogenic bacteria to the epithelial cells, thereby maintaining the integrity of the gut barrier (Son et al. 2022). Unlike proton pump inhibitors (PPIs), which can disrupt the gut microbiota and increase the risk of IBD, Tegoprazan provides a safer option for managing gastric acid without compromising gut health(Kim et al. 2021).

Addressing antibiotic-induced dysbiosis is another critical aspect of maintaining gut barrier integrity. Lentinan, a β-glucan derived from the macrofungus *Lentinus edodes*, has been shown to counteract the negative effects of antibiotics by boosting beneficial microbial species and promoting the production of short-chain fatty acids (SCFAs) (Zhou et al. 2024). These SCFAs play a vital role in nourishing intestinal epithelial cells and enhancing gut barrier function. In addition, incorporating dietary fibre from sources like the acacia plant before and after antibiotic exposure has been found to reduce E. coli colonisation in animal models. This suggests that specific plant fibres can protect the gut barrier by limiting the growth of pathogenic bacteria (Son et al. 2022).

Dietary strategies are also crucial for enhancing gut barrier integrity. Fermentable dietary fibre, also known as microbiota-associated carbohydrates, promotes the production of SCFAs, which are essential for the health of intestinal epithelial cells. A diet low in fibre has been linked to increased intestinal permeability and a higher abundance of harmful Proteobacteria, highlighting the protective role of dietary fibre (Holscher 2017; Nie et al. 2021). In addition, amino acids like glutamine and arginine, along with minerals such as zinc and vitamins A and D, contribute significantly to gut barrier integrity. Zinc, for example, influences TJ protein expression via the GPR39 receptor, while vitamins A and D modulate the expression of TJ proteins like occludin and ZO-1 ((Den Besten et al. 2013; García-Montero et al. 2021; Xiao et al. 2014; Zuhl et al. 2015).

Probiotics have emerged as a promising intervention for improving intestinal barrier integrity and limiting the development of inflammatory diseases. These live microorganisms, when administered in adequate amounts, confer health benefits on the host by enhancing the gut’s structural and functional integrity (Kaistha & Deshpande 2021). Probiotic strains, primarily from the genera *Lactobacillus* and *Bifidobacterium*, are involved in lowering intestinal pH, facilitating cell-to-cell signalling, preventing the colonisation of pathogenic microbes, and regulating the host immune response (Martín & Langella 2019; S. Wang et al. 2021). A subgroup of probiotics, termed “psychobiotics”, has been shown to improve psychological and mental health by influencing mood, anxiety, focus, memory, and cognition (Dinan et al. 2013; Sharma & Bajwa 2022).

Probiotics can modulate gut microbiota composition and restore the gut ecosystem, providing a potential approach for preventing and treating cognitive deficits (Min et al. 2022; Sivamaruthi et al. 2020). For instance, a probiotic mixture of *Lactobacillus acidophilus, L. rhamnosus*, and *Bifidobacteria longum* improved symptoms of autism after three months of supplementation (Dargenio et al. 2023; Sivamaruthi et al. 2020). Mechanisms underlying the effects of probiotics on the GBA include modulation of bacterial toxins and amyloid-beta interactions, pathways involving the vagus nerve, cytokines, and neurotransmitters, and impacts on the BBB and intestinal mucosal barrier integrity (Latif et al. 2023; Markowiak & Ślizewska 2017; Nettleton et al. 2021; Rose et al. 2021). Probiotics reduce oxidative stress by producing antioxidant enzymes and substances, such as catalase, superoxide dismutase, butyrate, folate, and glutathione, and chelating metal ions. They may prevent immune responses like inflammation by inhibiting TLR activation and enhancing BBB integrity (X. Wang et al. 2023). In addition, probiotics improve cognitive function in depression by reducing hypothalamic–pituitary–adrenal (HPA) axis dysfunction, increasing monoamine levels, and enhancing neuroplasticity (Ansari et al. 2020; Pinto-Sanchez et al. 2017; Rudzki et al. 2019).

Prolonged probiotic supplementation raises peripheral tryptophan levels, enhancing mental health by upregulating serotonin synthesis enzymes (Mills et al. 2023). Probiotics also modulate neurotransmitters such as serotonin, gamma-aminobutyric acid, acetylcholine, norepinephrine, dopamine, and glutamate, thereby regulating brain activity (Huang & Wu 2021; Lyte 2011; Meeusen & Roelands 2010). Probiotics, such as Bifidobacterium infantis, can restore the normal kynurenine-to-tryptophan ratio, demonstrating the potential of gut microbiota in modulating this pathway and maintaining gut barrier integrity (Purton et al. 2021; Underwood et al. 2015).

### Future Directions

The exploration of gut permeability, the GBA, the KP, and their implications for neurological disorders opens numerous avenues for future research. First, there is a need for longitudinal studies to establish causal relationships between gut permeability alterations and the onset of neurological disorders such as Alzheimer’s disease, Parkinson’s disease, and others. Such studies should include diverse populations to account for genetic and environmental variability (Cryan & Dinan 2012).

Further investigation into the molecular mechanisms linking gut permeability to systemic inflammation and neuroinflammation is crucial. This includes identifying specific bacterial strains and their metabolites that influence the KP and tight-junction integrity. Advanced omics technologies, including metagenomics, metabolomics, and proteomics, can provide comprehensive insights into the microbiome–host interactions and their impact on the CNS (O’Mahony et al. 2015).

Clinical trials focusing on therapeutic interventions targeting gut permeability and the KP are essential. Probiotics, prebiotics, and dietary interventions that support gut barrier function should be rigorously tested for their efficacy in preventing or mitigating neurological disorders. In addition, the development of specific inhibitors for enzymes in the KP, such as IDO and KMO, holds promise for reducing neurotoxic metabolite levels and improving neuronal health (Platten et al. 2019).

Investigating the role of the GBA in exercise physiology and its impact on gut permeability and inflammation is another promising area. Understanding how physical activity influences the gut microbiota and the KP could lead to novel strategies for enhancing cognitive function and mental health through exercise (Kennedy et al. 2017).

Lastly, personalised medicine approaches should be explored, considering individual variations in gut microbiota composition and genetic predispositions. Tailoring interventions based on a person’s microbiome profile and KP activity could optimise therapeutic outcomes and minimise adverse effects.

## Conclusion

Emerging evidence highlights the potential of targeting gut barrier integrity and the KP as novel therapeutic strategies for preventing and treating conditions such as Alzheimer’s disease, Parkinson’s disease, multiple sclerosis, and other neuropsychiatric disorders. By maintaining a balanced gut microbiota through diet, probiotics, and lifestyle modifications, and by developing specific inhibitors targeting the KP, it may be possible to mitigate neuroinflammation and neurodegeneration.

## Data Availability

No datasets were generated or analysed during the current study.

## Abbreviations

AD: Alzheimer’s disease
CNS: Central nervous system
EEC: Enteroendocrine cells
ENS: Enteric nervous system
GBA: Gut–brain axis
GI: Gastrointestinal
HPA: Hypothalamic–pituitary–adrenal
IA: Intestinal alkaline phosphatase
IDO: Indoleamine 2,3-dioxygenase
IEC: Intestinal epithelial cells
IECs: Intestinal epithelial cells
IL-1: Interleukin-1
IL-1β: Interleukin-1 beta
IL-6: Interleukin-6
IL-18: Interleukin-18
JAM-1: Junctional adhesion molecule 1
JNK: Jun N-terminal kinase
KP: Kynurenine pathway
KAT: Kynurenine aminotransferase
KMO: Kynurenine 3-monooxygenase
KYNA: Kynurenic acid
LPS: Lipopolysaccharide
MAPK: Mitogen-activated protein kinase
MS: Multiple sclerosis
MyD88: Myeloid differentiation primary response 88
NAD+: Nicotinamide adenine dinucleotide
NF-Κb: Nuclear factor kappa-light-chain enhancer of activated B cells
ND: Neurological disorder
NLRP3: Nucleotide-binding oligomerization domain, Leucine rich repeat and pyrin domain-containing 3
NSAIDs: Non-steroidal anti-inflammatory drugs
PD: Parkinson’s disease
PPIs: Proton pump inhibitors
QUIN: Quinolinic acid
SCFA: Short-chain fatty acids
STAT: Signal transducer and activator of transcription
TDO: Tryptophan 2,3-dioxygenase
TER: Transepithelial resistance
TJ: Tight junction
TLR: Toll-like receptor
TNF-α: Tumour necrosis factor-alpha
TRP: Tryptophan
ZO-1: Zonula occludens-1
ZO-2: Zonula occludens-2
ZO-3: Zonula occludens-3
